# Axial compression behavior of carbon fiber reinforced polymer confined partially encased recycled concrete columns

**DOI:** 10.1371/journal.pone.0304797

**Published:** 2024-06-03

**Authors:** Yunchen Wang, Jiongfeng Liang, Caisen Wang, Wei Li

**Affiliations:** 1 Faculty of Civil & Architecture Engineering, East China University of Technology, Nanchang, China; 2 Faculty of Architecture, Civil and Transportation Engineering, Beijing University of Technology, Beijing, China; 3 College of Civil and Architecture Engineering, Wenzhou University, Wenzhou, China; 4 Key Laboratory of Engineering and Technology for Soft Soil Foundation and Tideland Reclamation of Zhejiang Province, Wenzhou, China; Universiti Teknologi Malaysia, MALAYSIA

## Abstract

Partially encased concrete (PEC) has better mechanical properties as a structure where steel and concrete work together. Due to the increasing amount of construction waste, recycled aggregate concrete (RAC) is being considered by more people. However, although RAC has more points, the performance is inferior to natural aggregate concrete (NAC). To narrow or address this gap, lightweight, high-strength and corrosion-resistant CFRP can be used, also protecting the steel flange of the PEC structure. Therefore, carbon fiber reinforced polymer (CFRP) confined partially encased recycled coarse aggregate concrete columns were studied in this paper. With respect to different slenderness ratios, recycled coarse aggregate(RCA) replacement ratios, and number of CFRP layers, the performance of the proposed CFRP restrained columns are reported. The RCA replacement ratio is analyzed to be limited negative impact on the bearing capacity, generally within 6%. As for the slenderness ratio, the bearing capacity increased with it. However, wrapping CFRP significantly increased the bearing capacity. Considering the arch factor, a simple formula for calculating the ultimate strength of CFRP-confined partially encased RAC columns is developed based on EC4 and GB50017-2017. By comparison with the experimental values, the error is within 10%.

## 1. Introduction

Steel-concrete composite structure is assembled with steel and concrete to making good use of the performance of the two materials under the premise of ensuring both the steel and concrete work together. H-steel partially encased concrete (PEC) columns as one of the composite structure modes. Concrete was filled between the H-steel flanges, and the profile steel was used as longitudinal stress bar, stirrup and formwork in the concrete [1–3]. The stiffness, strength and member stability of the profile steel were reinforced by the filled concrete. This enabled PEC columns to surpass conventional steel structures in terms of mechanical properties [4, 5].

Recently, recycled aggregate concrete (RAC) saves natural resource by partly substituting natural aggregates with recycled aggregates and protects the living environment, and it is used as a green construction material. However, it has been found that RAC did not perform as well as natural aggregate concrete (NAC) [6–11]. Feng et al. [12] proposed waste rubber pellets modified recycled concrete and the results were also positive. There are a number of scholars have provided more experience regarding the improvement of strength by providing lateral restraint to concrete [13–16]. Besides that, confined concrete can also effectively improve the performance of RAC. Munir et al. [17, 18] investigated the strength of steel spiral-constrained recycled concrete through three different mix-ratio design tests, which showed that the strength of the restrained recycled concrete can approach the strength of natural aggregate concrete. In addition, Munir et al. [19] also developed a predictive model for the strength of spiral steel recycled concrete by machine learning. However, a more innovative method of confinement, external Carbon Fiber Reinforced Polymer (CFRP) had been deliberated among most scholars. CFRP is a lightweight, high strength material and easy to use for confinement [20, 21].

Many of existing researches have been performed to study the mechanical performance of RAC confined by steel tube or fiber reinforced polymer (FRP) [22, 23]. Wu et al [24] initiated an investigation about curing of RAC filled inside steel tubes. Taking into account internal curing, they also proposed a formula to calculate strength of recycle concrete filled steel tubes. Chen et al [25] explored 48 CFST with RAC columns and found the performance of the members were tiny affected by the replacement ratio of RCA. This result indicated that RACFSTs can be applied to practical engineering. Wang et al [26] discovered that the strength of the original waste concrete significantly affected the creep RAC-filled steel tubes, and the maximum deformation could vary by 37%. Nour and Güneyisi [27] explained the various parameter on the strength of RAC-filled tubes and developed a strength prediction model for RAC-filled tubes using programming techniques based on existing codes. The structural performance of RAC-filled steel tubes was analyzed by Xu et al [28] using and back-propagation neural network, and it was found that the parameters confining the steel tubes had the greatest impact on the performance of the RACFST. Additionally, a lot of researches focused on the performance of FRP confined RAC have been reported. Zhao et al. [29] used XGboost bypass mechanism analysis to develop a machine learning model for FRP constrained RAC reinforcement ratio. Numerous experimental data compared by Choudhury et al [30] and found that FRP improves the initial stiffness of RAC-filled tubes columns with significant dilatation. Gao et al [31] investigated RAC-filled tubes using recycled clay brick aggregates and found that CFRP improved their deformation and strength. The absence of size effect in CFRP confined RAC-filled tube columns was concluded by Chen et al who designed a series of tests. The use of RAC was also found to have no significant effect on FRP confined RAC [32]. GFRP reinforced RAC- filled tube columns were proposed by Tang et al [33], who found that the use of RAC can improve the ductility of the members.

The above literature review shows that there are more approaches on lateral constraint RAC. However, research on applying RAC to members and using the members themselves to provide ring confinement has mainly focused on the mechanical properties of steel tubes or FRP confined RAC columns. The PEC structure is a composite structure with excellent mechanical properties, while CFRP is lightweight, strong and easy to apply. To expand the structural application of confined RAC, this paper proposed an innovative confined RAC method- the CFRP confined partially encased RAC columns. To study the mechanical properties, all designed specimens were tested under concentric compression. The experimental phenomena during the compression process were observed and analyzed for performance with varying parameters. The bearing capacity calculations were carried out on the basis of the existing simplified formulae and the calculated values fitted very well with the test values.

## 2. Experimental program

### 2.1 Material properties

**Fig 1:**
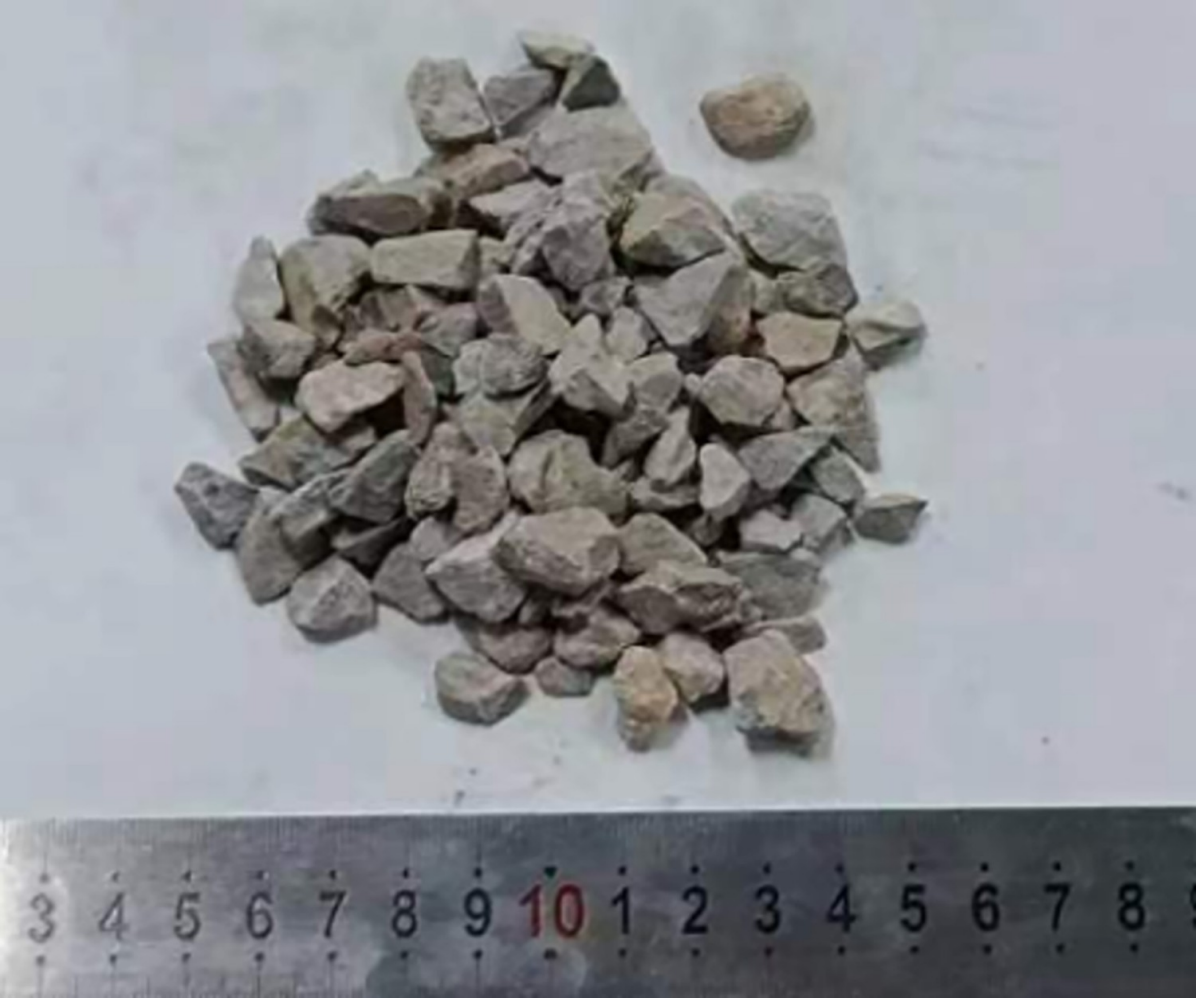
Natural coarse aggregate (NCA).

**Fig 2:**
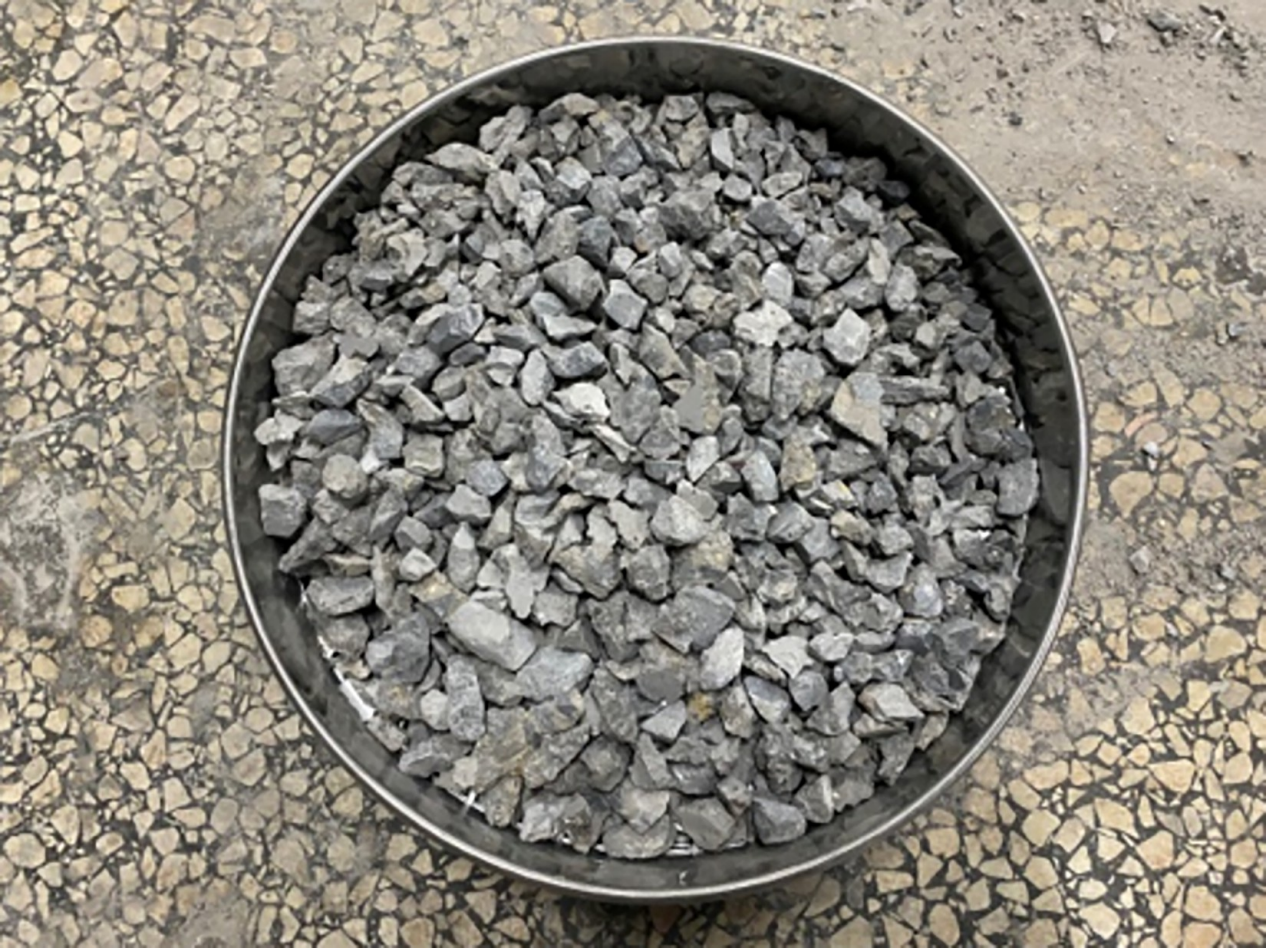
Recycled coarse aggregate (RCA).

**Fig 3:**
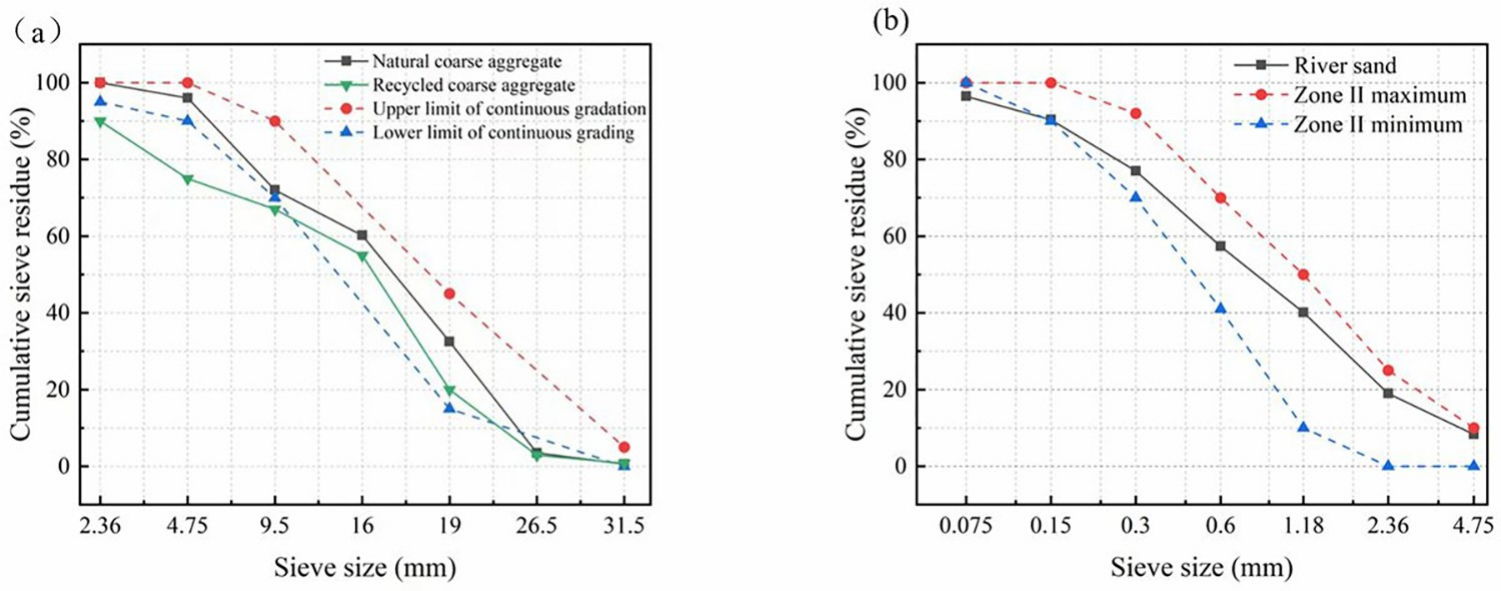
The gradation curve. (a) NAC and RAC, (b) River sand.

**Fig 4:**
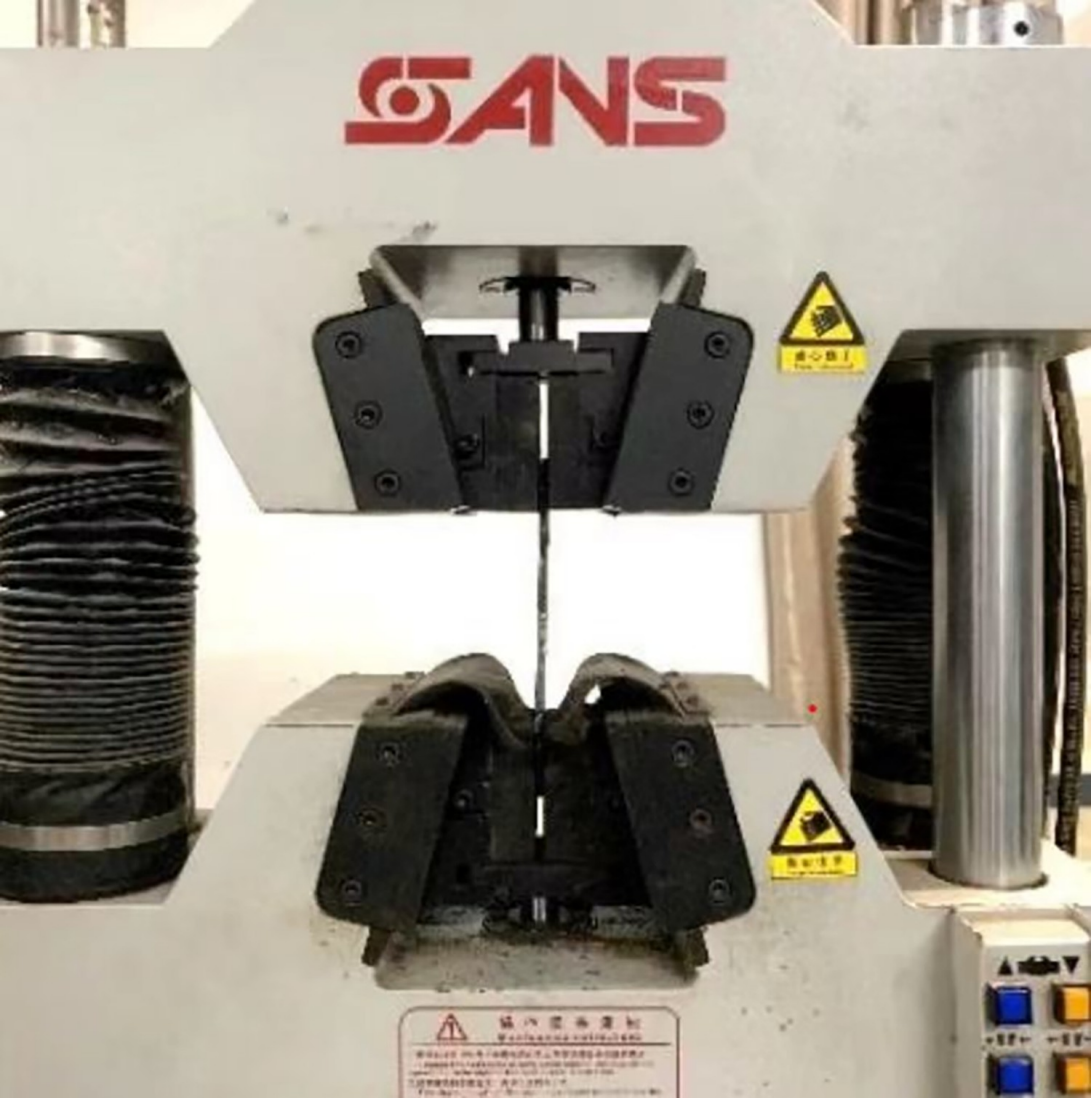
Steel testing.

For different sources of recycled aggregates, the mechanical properties of recycled concrete can have different or even opposite results [34, 35]. However, only recycled aggregates from the same type of construction waste were used in this paper. The coarse aggregates used for RAC in the test are shown in Figs 1 and 2. Among them, the recycled coarse aggregates came from waste concrete, and its performance satisfied GB/T25177-2010 [36]. The recycled coarse aggregate was soaked in water for 24h before preparing the concrete. Table 1 demonstrates the properties of coarse aggregates. Meanwhile, the grading curves of RCA, NCA and river sand are shown in Fig 3. According to the mix ratio in Table 2, different amounts of RCA were added while configuring the concrete. Table 2 shows the compressive strength (*f*_cu_) of cubes of RAC was tested according to GB/T50081-2002 [37]. As shown in the Fig 4, the properties of Q235 steel are tested listed in Table 3, in accordance with the specification GB/T 228–2010 [38].

**Table 1:** Properties of the aggregates.

Aggregate	Specific gravity	Specific gravity	Water absorption	Chloride Content
	(SSD)	(oven dried)	%	(% of cement mass)
NCA	2.86	2.84	0.87	0.13
RCA	2.39	2.31	3.55	0.29

**Table 2:** Mix ratio of RAC.

Type	ID	*r*(%)	RCA	Cement	Sand	NCA	Water	*f*_cu_(MPa)	Curing Time
			(kg·m^-3^)						
C30	C30-0	0	0	513	474	1218	195	33.1	28d
	C30-50	50	609	513	474	609	195	28.9	
	C30-100	100	1218	513	474	0	195	26.2	
C40	C40-0	0	0	454	595	1152	200	41.8	
	C40-50	50	576	454	595	576	200	38.3	
	C40-100	100	1152	454	595	0	200	35.7	

Note: *r*-the recycled aggregate replacement ratio.

**Table 3:** Mechanical properties of steel.

Type	$\begin{array}{c} Tensile\;yield\;stress\\ \left(MPa\right) \end{array}$	$\begin{array}{c} Ultimate\;tensile\;stress\\ \left(MPa\right) \end{array}$	$\begin{array}{c} Elastic\;modulus\\ \left(GPa\right) \end{array}$	Tensileyieldstrain
Flange	247	282	204	1221
Web	246	288	201	1228

Epoxy resin adhesive is used to attach CFRP to the surface of PEC columns. The epoxy resin adhesive is an AB adhesive, which needs to be prepared according to 1:2 mix when used. Before wrapping, the surface of the specimens was cleaned and the CFRP was cut according to the design. After configuring the epoxy adhesive in proportion, applying the glue evenly on the surface of CFRP and specimens, and pressed evenly to paste. After construction, carry out maintenance and curing. The CFRP sheet used in this test was 100 mm width and 0.167 mm thickness and other material parameters were provided by the manufacturer as presented in Table 4.

**Table 4:** Parameters for CFRP.

Type	$\begin{array}{c} Ultimate\;tensile\;strength\\ \left(MPa\right) \end{array}$	$\begin{array}{c} Tensile\;strain\;at\;fracture\\ \left(\%\right) \end{array}$	$\begin{array}{c} Elastic\;modulus\\ \left(GPa\right) \end{array}$
CFRP	3471MPa	2.1	255

### 2.2 Specimen design

**Fig 5:**
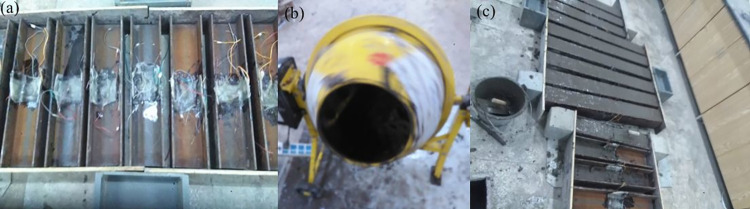
Specimens manufacturing process. (a) Steel, (b) Preparation of concrete, (c) Pouring concrete.

**Fig 6:**
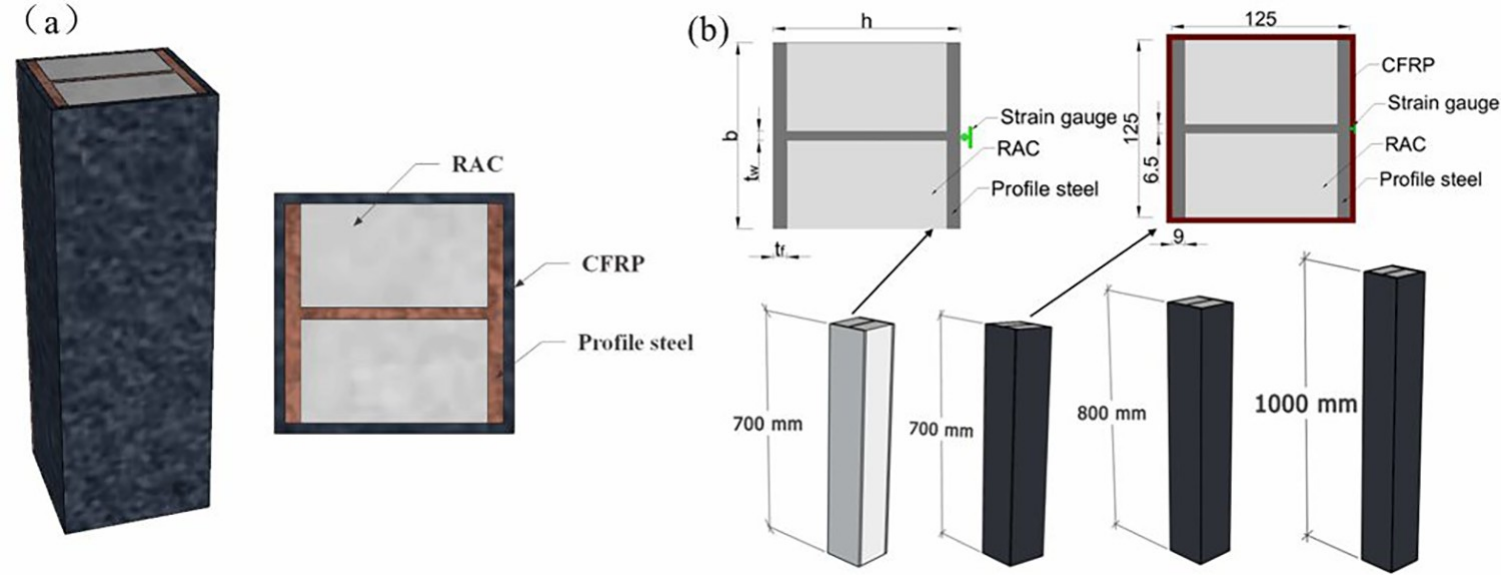
Schematic view of CFRP confined columns. (a) Three-dimensional drawing of specimen, (b) Size of specimens.

In the experiment, 14 CFRP confined partially encased RAC columns were designed. The cross-section size of H-steel was 125mm×125mm, and the thickness of web and flange plates were 6.5mm and 9mm, respectively. Detailed parameters were shown in Table 5. After the H-steel was fabricated by the fabricator, it was poured. One side was poured at a time, and the second side was poured after a few days of maintenance, as shown in Fig 5. The sketch of the CFRP partially encased RAC column shown in Fig 6. The following variables were tested herein:

- Recycled coarse aggregate replacement ratio (*r*): 0,50%,100%
- Number of CFRP sheet layers (*n*): 0, 1,2
- Design concrete strength (*f*_cu,d_): C30,C40
- Slenderness ratio (*λ*): 5.6, 6.4, 8

**Table 5:** Design parameters of the specimens.

Specimen	*h*×*b*×*t*_w_×*t*_f_(mm)	*L*	*r*(%)	*n*	*f*_cu,d_	λ
		(mm)				(*L/b*)
Z5-0-1-30	125×125×6.5×9	700	0	1	C30	5.6
Z5-50-1-30	125×125×6.5×9	700	50	1	C30	5.6
Z5-100-1-30	125×125×6.5×9	700	100	1	C30	5.6
Z6-0-1-30	125×125×6.5×9	800	0	1	C30	6.4
Z6-50-1-30	125×125×6.5×9	800	50	1	C30	6.4
Z6-100-1-30	125×125×6.5×9	800	100	1	C30	6.4
Z8-0-1-30	125×125×6.5×9	1000	0	1	C30	8.0
Z8-50-1-30	125×125×6.5×9	1000	50	1	C30	8.0
Z8-100-1-30	125×125×6.5×9	1000	100	1	C30	8.0
Z5-100-0-30	125×125×6.5×9	700	100	0	C30	5.6
Z8-100-2-30	125×125×6.5×9	1000	100	2	C30	8.0
Z5-0-1-40	125×125×6.5×9	1000	0	1	C40	5.6
Z6-50-1-40	125×125×6.5×9	1000	50	1	C40	6.4
Z8-100-1-40	125×125×6.5×9	1000	100	1	C40	8.0

The label “Z5-100-1-30”represents the slenderness ratio is 5.6, the RCA replacement rate is 100%, the CFRP is 1 layer, the concrete strength is C30.

### 2.3 Loading method

**Fig 7:**
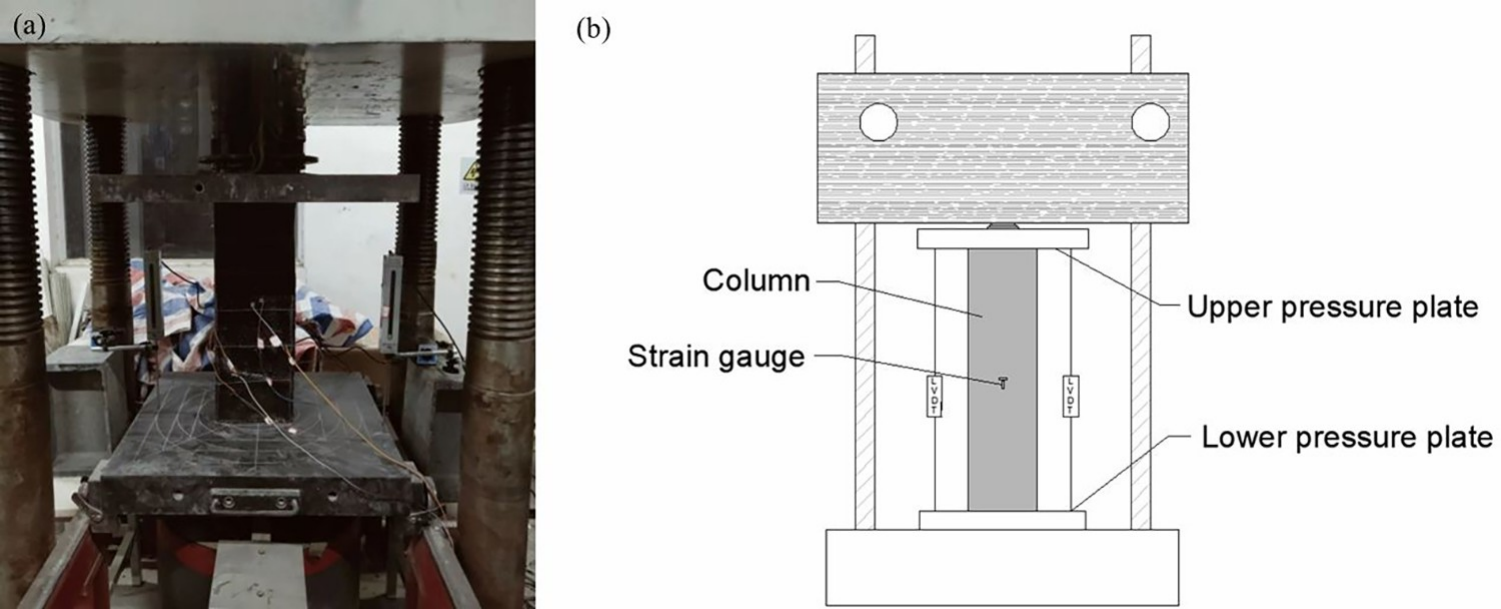
Test setup. (a) loading chart, (b) Loading Sketch.

As displayed in Fig 7, all specimens were tested in concentric compression by load and displacement control. The front end was loaded at a rate of 5 kN/s up to 0.9 *N*_max_. Finally, a displacement rate of 0.03 mm/s was applied until the load was reduced to 0.8 *N*_max_. The front end was loaded at a rate of 5 kN/s up to 0.9 *N*_max_.

## 3. Experimental results

### 3.1 Failure patterns

**Fig 8:**
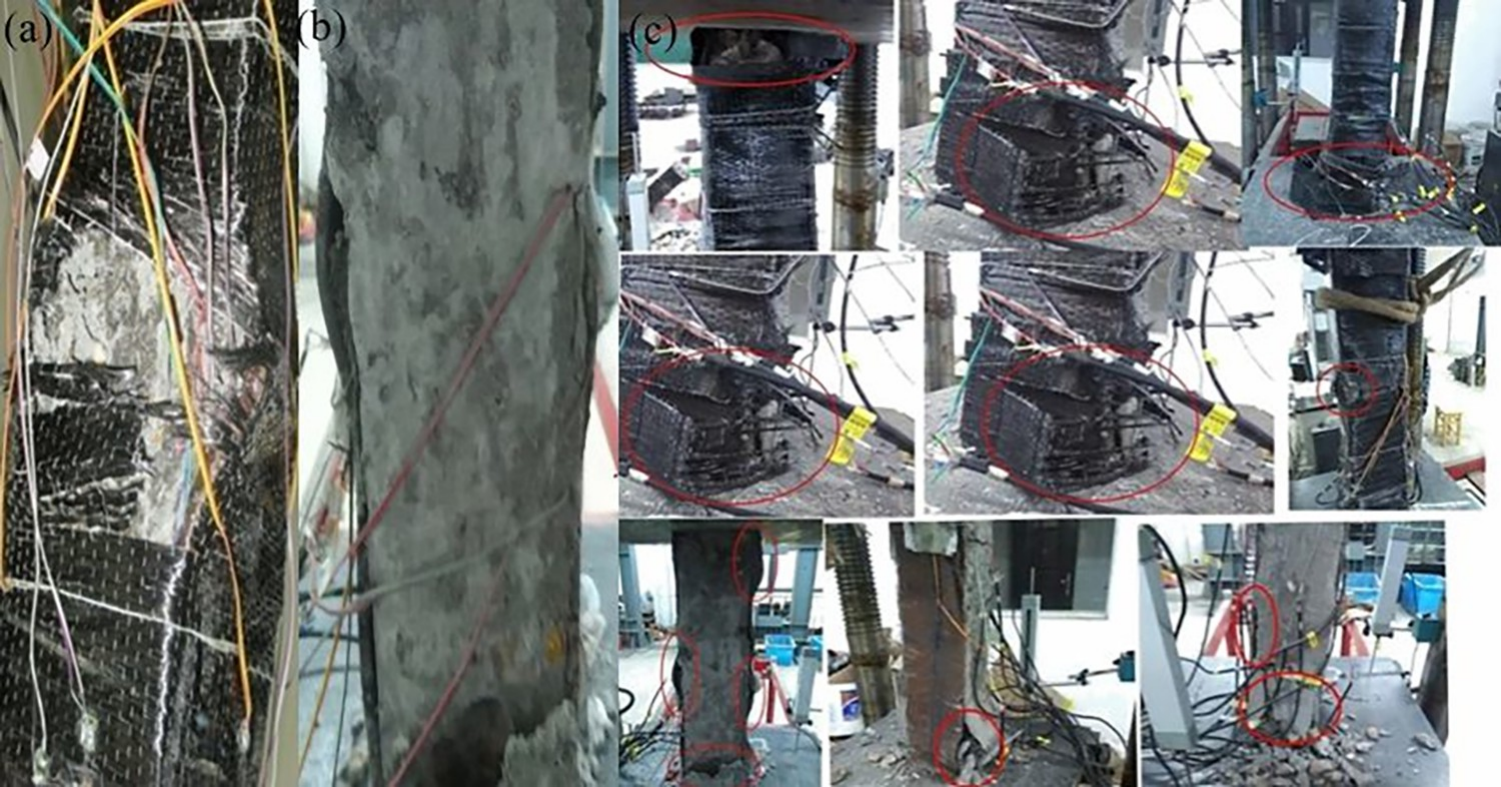
Failure state of specimens. (a) Confined CFRP, (b) Unconfined CFRP, (c) Failure details of specimens.

The damage to the CFRP sheet-wrapped specimens was mainly strength damage. Specimen failure was characterized by surface CFRP fracture and steel flange buckling. No obvious changes, including cracking and deformation, were observed in the CFRP confined columns during the initial loading stage. After loading for a while, the resin makes an incessant sound. When the applied load was 0.8*N*_max_, white drawing appeared on the outer surface of CFRP. As the loading was continued, the CFRP at the corners of the specimens suddenly started to fracture, reached 0.9*N*_max_. For the PEC columns unconfined CFRP, the damage pattern manifested as local buckling of the steel flange of both ends. Fig 8 exhibited a typical damage patterns. The concrete was damaged or even dislodged.

### 3.2 Load-displacement relationships

**Fig 9:**
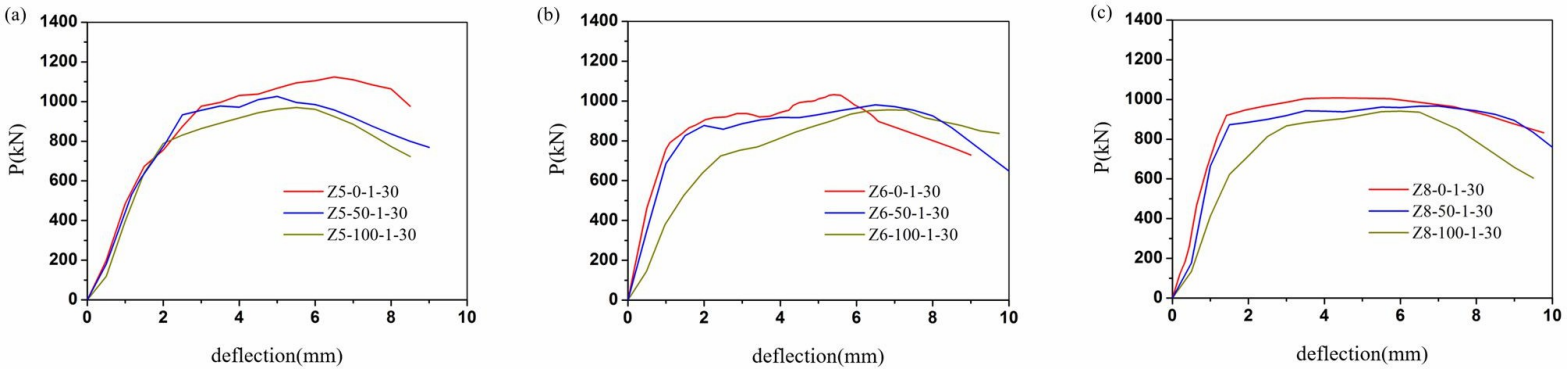
Load-displacement relationships of different recycled aggregate replacement percentage. (a)Z5, (b)Z6, (c)Z8.

**Fig 10:**
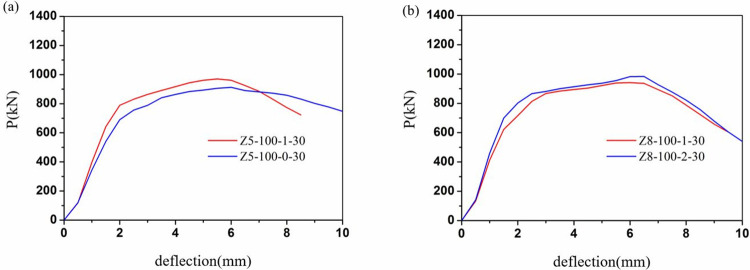
Load-displacement relationships of different thickness of CFRP sheet. (a)Z5-100, (b)Z8-100.

**Fig 11:**
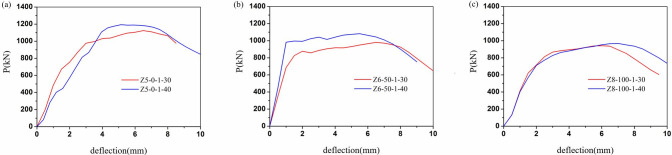
Load-displacement relationships of different concrete strength. (a)Z5-0, (b)Z6-50, (c)Z8-100.

**Fig 12:**
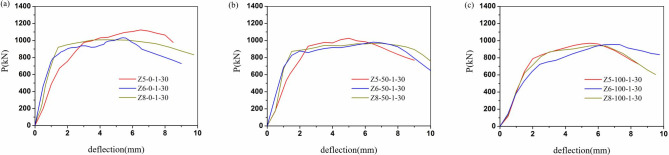
Load-displacement relationships of different slenderness ratio. (a)ZX-0, (b)ZX-50, (c)ZX-100.

The load-displacement relationships of all columns were illustrated in Figs 9–12. It can be evident that the load-displacement relationships of all the columns exhibited a similar behavior. And the typical load-displacement relationships could be classified into three phases, i.e., elastic, elastoplastic and plastic [39]. In the elastic phase, the load-displacement relationships were the linear responses. As about 60% of the peak strength, the load-displacement relationships demonstrated non-linear responses at the elastoplastic phase. After the load achieved peak load, the load-displacement relationships entered the plastic stage and the load dropped along with the curves bend down due to the rupture of the CFRP.

Fig 9 illustrated the load-displacement relationship for different recycled aggregate replacement ratios. From the Fig 9, the curve with large slenderness ratio was generally located below. This meant that the larger the replacement ratio of RA, the performance such as stiffness and bearing capacity showed a decreasing trend. Due to the fact that recycled aggregates inherently have certain defects in mechanical properties. In particular, the slope of the rising section of the curve was significantly minimized at 100% replacement of RCA for large length to slenderness ratios.

The load-displacement curves for different number of CFRP layers were shown in Fig 10. For the same conditions, the specimens wrapped with 1 layer of CFRP showed a greater slope in the rising section of the curve and a higher peak load than the unwrapped specimens. Similarly, for the Z8-100 series, the load-displacement curves for the 2-layer were generally higher than those for the 1-layer. The wrapping of the CFRP sheet provided a lateral restraint by delaying yielding of the flange and the damage to the concrete.

Fig 11 displayed the variation of load displacement curves with concrete grade. The greater the strength of concrete at recycled aggregate replacement ratio not more than 50%, the steeper the curve at the beginning of the curve and the greater the peak load. In other words, the increasing concrete strength improved the initial stiffness and bearing capacity of the specimens at 0–50% replacement of RCA.

In Fig 12, the slope of the ascending segment increased and then decreased with increasing slenderness ratio in the same case. However, the effect of slenderness ratio seemed to be very small as the replacement ratio of RCA was 100%. It meant that the impact of slenderness ratio on peak load and stiffness was limited at 100% replacement of RCA.

### 3.3 Load carrying capacity

**Fig 13:**
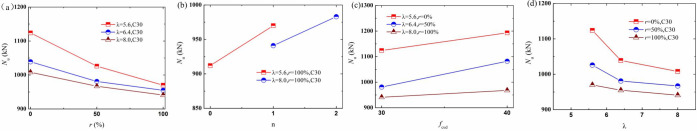
Load carrying capacity with different parameters. (a) Recycled coarse aggregate replacement percentage, (b) Number of CFRP sheet layers, (c) Concrete strength, (d) Slenderness ratio.

Table 6 and Fig 13 have shown bearing capacity of the specimens for different parameters. A reduction in the bearing capacity of the specimens can be observed as the replacement ratio of RCA increased. This is similar to the findings of Cai et al [40]. Compared with that of specimen Z5-0-1-30 with NCA, the bearing capacity of specimen Z5-100-1-30 with 100% RCA was 13.8% lower.

**Table 6:** Experimental results of the specimens.

Specimen	*N*_u_ (kN)	*Δ*_u_(mm)	*Δ*_0.85_ (mm)	*SI*	*DI*
Z5-0-1-30	1124	6.5	9.35	1.00	1.44
Z5-50-1-30	1026	5.0	7.16	0.91	1.43
Z5-100-1-30	970	5.5	7.52	0.86	1.37
Z6-0-1-30	1039	4.8	6.76	0.92	1.41
Z6-50-1-30	981	6.5	8.74	0.87	1.34
Z6-100-1-30	955	6.8	7.88	0.85	1.16
Z8-0-1-30	1008	4.4	9.36	0.89	2.13
Z8-50-1-30	967	7.0	9.58	0.86	1.37
Z8-100-1-30	941	6.0	7.93	0.84	1.32
Z5-100-0-30	912	6.0	9.52	0.81	1.59
Z8-100-2-30	983	6.5	7.89	0.87	1.21
Z5-0-1-40	1193	5.1	8.42	1.06	1.65
Z6-50-1-40	1082	5.5	7.85	0.96	1.43
Z8-100-1-40	968	7.0	9.28	0.86	1.33

From Fig 13(B), the strength of the specimens has been raised due to the wrapping of CFRP. The bearing capacity of specimen Z5-100-1-30 was 19.9% higher than that of specimen Z5-100-0-30. Compared with specimen Z8-100-1-30, the bearing capacity of specimen Z8-100-2-30 was 4.5% higher. This demonstrated that CFRP provided lateral restraint and postponed the localized deformation of the steel flange, so increasing the bearing capacity. Compared with specimen Z6-50-1-30, the bearing capacity of specimen Z6-50-1-40 was 10.3% higher, as shown in Fig 13(C). T It was due to the greater the concrete strength contributed more bearing capacity at the same situation. Compared to specimen Z5-50-1-30 with a slenderness ratio of 5.6, the bearing capacity of specimen Z8-50-1-30 with a slenderness ratio of 8.0 was 5.8% lower, as shown in Fig 13(D). It showed that too large a slenderness ratio would, on the contrary, make the load carrying capacity decrease.

### 3.4 Strength index

To evaluate the effect of CFRP sheet on the strength improvement of columns, the improvement index (SI) was defined as:

$$SEI=\frac{N_{u}}{N_{Z5-0-1-30}}$$ (1)

Where *N*_u_ and *N*_*Z*5−0−1−30_ were the ultimate strength of specimens and that of specimen Z5-0-1-30, respectively.

**Fig 14:**
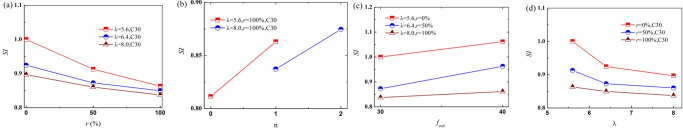
Strength index with different parameters. (a) Recycled aggregate replacement percentage, (b) Number of CFRP sheet layers, (c) Concrete strength, (d) Slenderness ratio.

Table 6 and Fig 14 displayed the strength index with different parameters. It is observed that SI decreased with incremental RCA replacement ratio. The SI of specimens Z5-50-1-30 with 50% RCA and Z5-100-1-30 with 100% RCA decreased by 9% and 14% compared to specimen Z5-0-1-30 with NCA, respectively. The SI of specimens Z6-50-1-30 with 50% RCA and Z6-100-1-30 with 100% RCA decreased by 5% and 8% compared to specimen Z6-0-1-30 with NCA, respectively. The SI of specimens Z8-50-1-30 with 50% RCA and Z8-100-1-30 with 100% RCA decreased by 3% and 5% compared to specimen Z8-0-1-30 with NCA, respectively.

As observed in Fig 14(B), the SI improved with increasing CFRP sheet layer number. The improvement of SI for the column with 100% RCA, a slenderness ratio of 5.6 and concrete strength grade of C30 was 19% when the CFRP sheet layer number increased 0 to 1.And compared to column Z8-100-1-30 with one CFRP sheet layer, the SI of column Z8-100-2-30 with two CFRP sheet layers improved 4%. This reveals that the CFRP provided effectively confining lead to enhancement in load-carrying capacity.

From Fig 14(C), as the strength of the concrete increased, so did the SI. The SI of specimen Z5-0-1-40 with C40 increased by 6% compared with that of specimen Z5-0-1-30 with C30.And the SI of specimen Z6-50-1-40 with C40 increased by 9% compared with that of specimen Z6-50-1-30 with C30.The SI of specimen Z8-100-1-40 with C40 increased by 3% compared with that of specimen Z8-100-1-30 with C30.

From Fig 14(D), the SI declined with the growth of the slenderness ratio. The SI of specimens Z6-50-1-30 with a slenderness ratio of 6.4 and Z8-50-1-30 with a slenderness ratio of 8.0 reduced 4% and 5% compared to specimen Z5-50-1-30 with a slenderness ratio of 5.6, respectively. And the SI of specimens Z6-100-1-30 with a slenderness ratio of 6.4 and Z8-100-1-30 with a slenderness ratio of 8.0 declined 2% and 3% compared to specimen Z5-100-1-30 with a slenderness ratio of 5.6, respectively.

### 3.5 Ductility index

To study the ductility property of CFRP confined partially encased RAC columns, a ductility index (DI) can be defined as follows:

$$DI=\frac{\Delta_{0.85}}{\Delta_{u}}$$ (2)

Δ_0.85_-the displacement at 0.85*N*_max_ (descending segment), Δ_u_-the displacement at *N*_max_.

**Fig 15:**
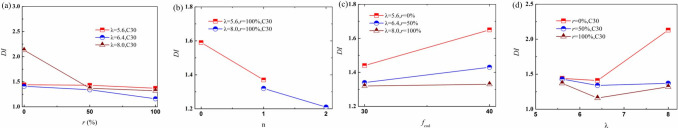
Ductility index with different parameters. (a) Recycled aggregate replacement percentage, (b) Number of CFRP sheet layers, (c) Concrete strength, (d) Slenderness ratio.

Table 6 and Fig 15 listed the DI of all specimens. It was observed that DI declined slightly as the RCA replacement rate increased. Nevertheless, as the number of layers of CFRP increased, the DI value also went up [41]. Additionally, DI augmented as the concrete strength increased. As the slenderness ratio grew from 5.6 to 8.0, DI increased and then decreased.

### 3.6 Stress-strain

**Fig 16:**
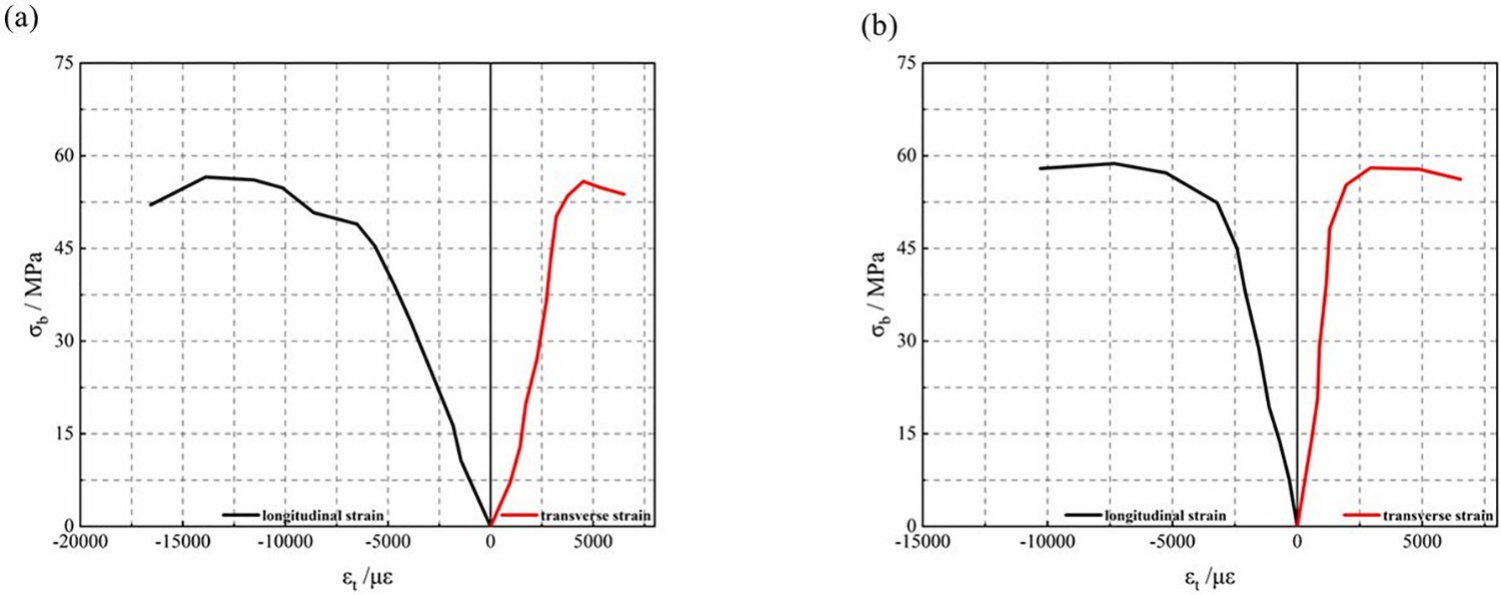
Stress-strain curves of specimens. Z5-100-0-30, Z8-100-2-30.

The stress-strain relationship of the steel flange was illustrated in Fig 16. The strain of specimen Z5-100-0-30 initially varied very little due to the fact that it was not wrapped with CFRP. As the test progressed, the concrete cracked and even crushed. This put a squeezing force on the steel flanges to the outside, resulting in increased deformation of the flanges. Thus later in the curve, the strain entered a phase of rapid growth. For the Z8-100-2-30 specimen, the rate of change of the beginning deformation is smaller than that of the unwrapped specimen. This was due to the fact that the presence of CFRP was able to inhibit the deformation of the steel flanges as well as retard the destruction of the concrete. However, as the load increases, the fracture of the CFRP caused the steel flange to deform rapidly. This also caused the curve to become very flat and the strain to increase rapidly.

## 4. Calculation of ultimate load-carrying capacity

**Fig 17:**
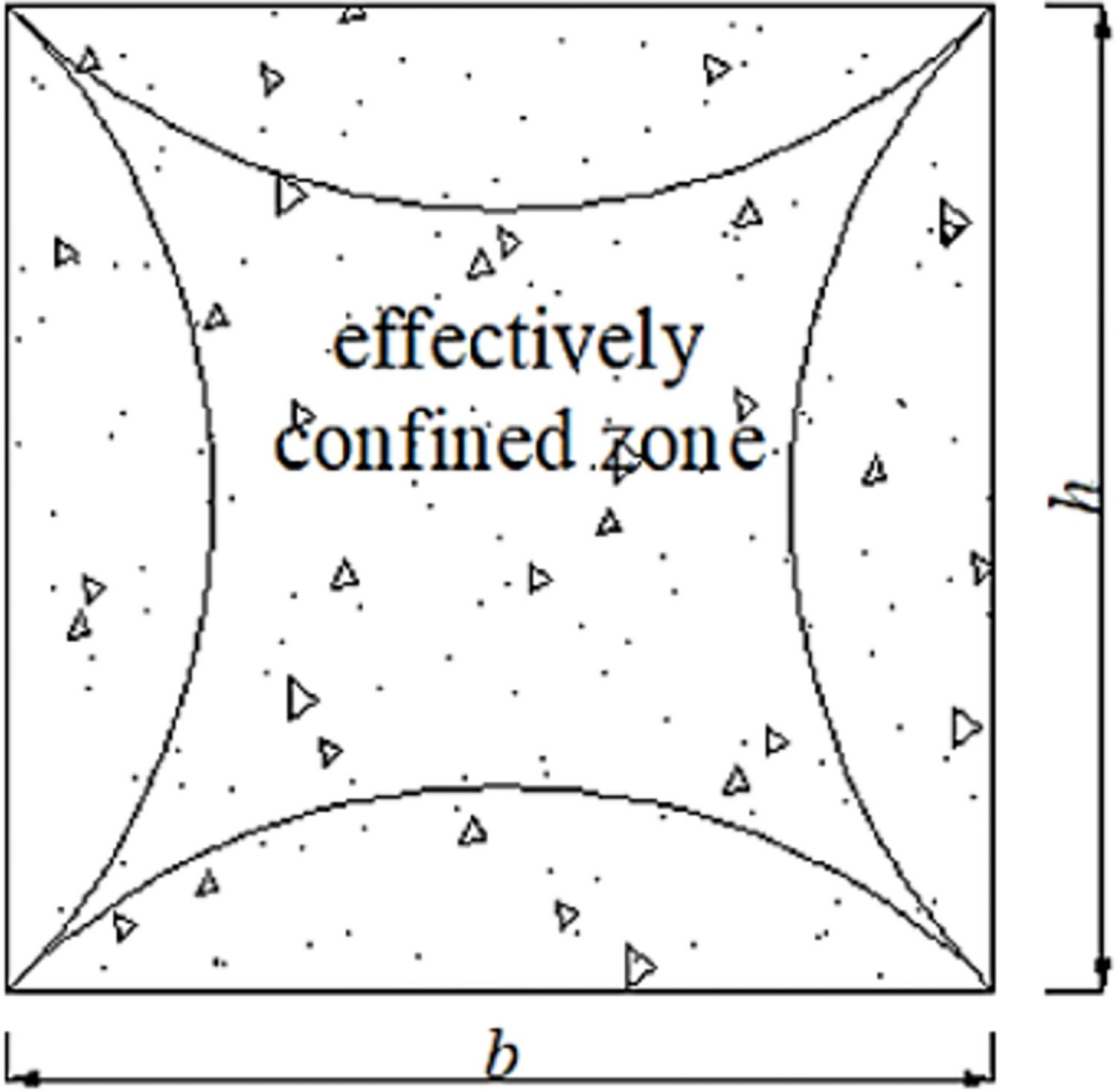
FRP-confined square cross-section.

FRP confined square cross-section is different from confined circular cross-section, Lam and Teng [42] proposed "arching effect", as shown in the Fig 17. The region consisting of four parabolas is the effective confined zone. However, the lateral restraint(*f*_lu_) can be expressed,

$$f_{lu}=\frac{2f_{frp}t_{frp}}{D}$$ (3)

$$D=\sqrt{h^{2}+b^{2}}$$ (4)

By using the EC4 (2004) codes [43], Liang [41] raised an equation to predict the ultimate strength of CFRP confined PEC columns. The buckling factor (*φ*) can be get from GB50017-2017 [44].


$$N=\varphi (A_{a}f_{a}+0.85A_{c}{f^{'}}_{cc})$$ (5)

$$f_{cc}^{'}=f_{co}^{'}+2.0f_{lu}$$ (6)

$$f_{lu}=\frac{2f_{frp}t_{frp}}{\sqrt{h^{2}+b^{2}}}$$ (7)

*A*_*a*_, and *A*_*c*_-the cross-section area of H-steel and concrete; *f*_*a*_-the yield strength of steel; $f_{cc}^{'}$ and $f_{co}^{'}$-compressive strength of confined and unconfined concrete; *f*_*frp*_- the tensile strength of FRP; *t*_*frp*_ - the thickness of FRP; *D—*equivalent diameter; *h* and *b*-the height and width of the section, respectively.

**Fig 18:**
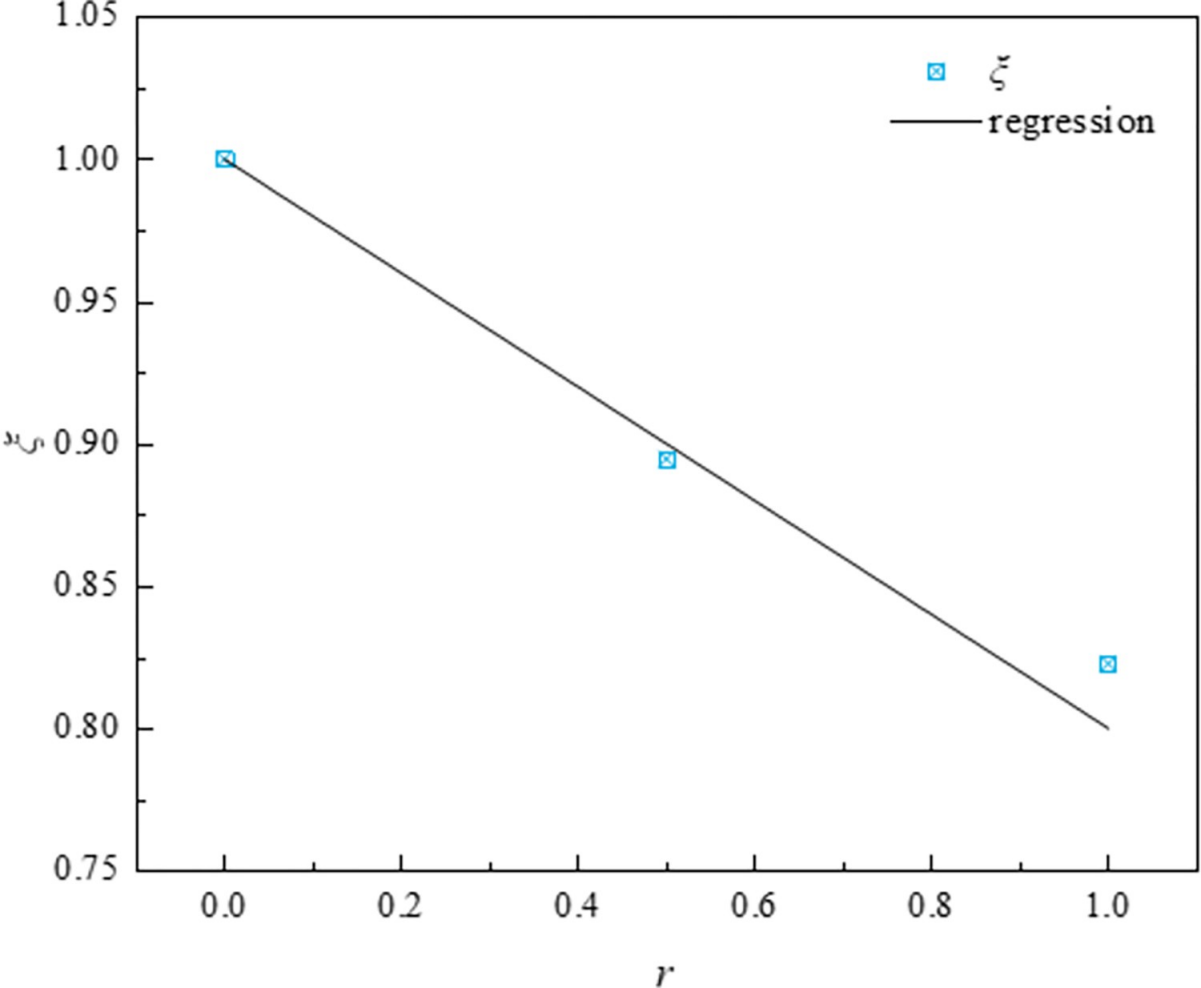
Regression curve of the discount factor.

Considering the influence of recycled coarse aggregate on concrete strength, based on the experimental data, the relationship between the replacement rate(*r*) of recycled aggregate and the factor (*ξ*) affecting concrete strength was obtained as follow, and the curve as shown in the Fig 18.


$$\xi =\frac{5-r}{5}$$ (8)

Therefore the ultimate strength of CFRP confined partially encased RAC columns is calculated as,

$$N_{u}=\xi \varphi A_{a}f_{a}+0.85\varphi A_{c}{f^{'}}_{cc}$$ (9)

The results of test and calculated based on the equation were compared in Table 7. Generally speaking, the calculated results agree well with the test results of composite columns. Therefore, the equation can be used to evaluate the CFRP confined partially encased RAC columns.

**Table 7:** Comparison of tested and calculated results.

Specimen	Test results (kN)	Calculated results(kN)	Error (%)
Z5-0-1-30	1124	1114	0.90
Z5-50-1-30	1026	968	5.99
Z5-100-1-30	970	890	8.94
Z6-0-1-30	1039	1101	-5.63
Z6-50-1-30	981	957	2.47
Z6-100-1-30	955	880	8.48
Z8-0-1-30	1008	1074	-6.15
Z8-50-1-30	967	933	3.63
Z8-100-1-30	941	858	9.61
Z5-100-0-30	912	834	9.35
Z8-100-2-30	983	968	1.58
Z5-0-1-40	1193	1183	0.85
Z6-50-1-40	1082	1022	5.90
Z8-100-1-40	968	919	5.34

## 5. Conclusions

Analyzing the test data of the all columns, the following conclusions can be drawn:

1. The failure mode of the specimens exhibited CFRP fracture and steel flange buckling. CFRP fracture occurred at corners due to stress concentration.
2. In this test, the bigger the replacement ratio of RCA, the poorer the bearing capacity. However, at 50%-100% replacement, the varying carrying capacity was within 6%. As for the slenderness ratio, the bearing capacity increased with it.
3. For ductility, at recycled aggregate replacement ratio of 0, the specimen with slenderness ratio of 8 improved 47.9% than that with slenderness ratio of 5.6. However, the effect of slenderness ratio on ductility was not tiny when the RA replacement ratio was 50%-100%.
4. The load-displacement curves of the specimens were generally divided into: elastic, elastoplastic, and plastic phases.
5. It was found that the calculated equation of the bearing capacity for CFRP confined PEC columns was acceptable for the calculation of that of CFRP confined partially encased RAC columns.
6. The effect of various wrapping schemes of CFRP (e.g., spaced wrapping) on the load carrying capacity of the specimens can also be considered in future studies.
7. The proposed object of this paper can be applied to the reconstruction project of the original building. It can save the cost by using local materials and also protect the environment.
8. In this paper, we mainly consider circumferential full wrapping, and partial wrapping and other angular wrapping methods can be further investigated in the future.

## Supporting information

S1 Data (XLSX)
